# Validating Indigenous Farmers’ Practice in the Management of the Fall Armyworm *Spodoptera frugiperda* (J. E. Smith) in Maize Cropping Systems in Africa

**DOI:** 10.3390/life14020180

**Published:** 2024-01-25

**Authors:** Saliou Niassy, Evanson Rigan Omuse, John Emanuel Khang’ati, Ines Bächinger, David Mfuti Kupesa, Xavier Cheseto, Benjamin W. Mbatha, Robert S. Copeland, Samira Abuelgasim Mohamed, Mphatso Gama, Joyce Mulila Mitti, Yeneneh Belayneh, Nicolas Delabays, François Lefort, Sunday Ekesi, Sevgan Subramanian

**Affiliations:** 1 International Centre of Insect Physiology and Ecology, Nairobi P.O. Box 30772-00100, Kenya; eomuse@icipe.org (E.R.O.); jkhangati@icipe.org (J.E.K.); dkupesa@icipe.org (D.M.K.); xcheseto@icipe.org (X.C.); bmbatha@icipe.org (B.W.M.); rcopeland@icipe.org (R.S.C.); sfaris@icipe.org (S.A.M.); sekesi@icipe.org (S.E.); ssubramania@icipe.org (S.S.); 2 Research Institute Land Nature Environment, Geneva School of Engineering Architecture and Landscape, HES-SO University of Applied Sciences and Arts Western Switzerland, CH-1254 Jussy, Switzerland; ibaechinger@gmail.com (I.B.); nicolas.delabays@hesge.ch (N.D.); 3 Machinga Agricultural Development Division, Liwonde Private Bag 3, Malawi; samu.gama@agriculture.gov.mw; 4 Figtree Limited (Figtree Consulting Services), Lusaka P.O. Box 33304-10101, Zambia; joyce.mulilamitti@fao.org; 5 USAID’s Bureau for Humanitarian Assistance (BHA) and Technical and Program Quality (TPQ), 1300 Pennsylvania Avenue, Washington, DC 20523, USA; ybelayneh@usaid.gov

**Keywords:** agroecology, integrated pest management, volatile organic compounds, smallholder farmer

## Abstract

Before the invasion of the fall armyworm (FAW) *Spodoptera frugiperda* into Africa, smallholder farmers had been using indigenous practices such as applying fish soup to plants to manage stemborer pests. Although farmers have since begun adapting this practice against FAW, no attempt has been made to scientifically evaluate this practice. Therefore, we assessed the efficacy of applying fish soup to maize plants that were artificially infested with FAW under semi-field conditions. Our results showed that foliar damage is inversely correlated with the concentration of a fish soup and sugar solution, with the highest (100%) concentration resulting in the lowest foliar damage and the highest plant recovery. The FAW foliar damage results for maize plants treated with 100%, 50%, 10% fish soup and sugar, and distilled water were 46.3 ± 5.6, 51.1 ± 5.0, 71.6 ± 5.2, and 99.4 ± 0.4%, respectively, whereas plant recovery results from the same treatments were 35.2 ± 3.7, 31.1 ± 5.4, 20.0 ± 4.6, and 0.0 ± 0.0%, respectively. A concentration of fish soup and sugar solution of at least 25.9% was required to achieve the lowest foliar damage of 17.8% and peak plant recovery of 73.6%. Fish soup and sugar solutions attracted a wide range of insects, including potential natural enemies (predators and parasitoids) of FAW, in a dose-dependent manner. Maize plants treated with fish soup and sugar showed higher chlorophyll content and better growth than the control did. Proximate and chemical analysis showed that fish soup contains essential plant growth nutrients (e.g., nitrogen, phosphorus, and calcium). Through GC-MS analyses, we identified 76 volatile organic compounds in fish soup, of which 16 have been reported as insect attractants, highlighting their potential ecological significance. Therefore, the indigenous pest management practices for FAW, such as the use of fish soup, deserve particular attention. These practices could contribute to food security and improve the livelihoods of vulnerable communities. Further field validation studies, economic analyses, product development, and optimisation are therefore required to optimise the use of fish soup based on fish waste.

## 1. Introduction

The fall armyworm (FAW), *Spodoptera frugiperda* (J. E. Smith), is an invasive pest originating from the Americas and was first reported in Africa in early 2016 [1,2]. FAW is a highly polyphagous pest and feeds on at least 350 plant species belonging to over 27 families [3]. However, Poaceae, like maize, sorghum, rice, wheat, and sugar cane, are the most preferred hosts of FAW [1]. Moths of FAW actively move at night to locate hosts on which to lay their eggs. Additionally, instar larvae 1 to 3 have an initial cryptic feeding behaviour on host plants, hiding their presence in plant whorls [4,5]. FAW is also reproductively very efficient in tropical areas, where the warmer temperatures allow for more generations to be propagated per year, compared with temperate areas that may have one up to two in a year [6,7]. Therefore, seasonal infestations of maize crops have been reported in most African countries because about 92% of the area under maize farming in Africa supports the conditions for year-round survival and reproduction of FAW [8].

FAW is a new burden for most smallholder cereal farmers in sub-Saharan Africa (SSA). For instance, crops in SSA, worth over 13 billion USD, are at risk of FAW damage, thereby threatening the livelihoods of millions of smallholder farmers and posing a serious threat to food security [9,10]. Chemical insecticides are the primary control strategy; however, this strategy is unsustainable [11,12], and most of the insecticides are either not affordable or inaccessible to smallholder farmers [13]. Continuous use of chemical insecticides can induce resistance in FAW populations [14,15]. Furthermore, most chemical insecticides are broad-spectrum, and their injudicious use is associated with several human and environmental health concerns [16]. Therefore, there is a need to develop and promote affordable, accessible, and environmentally friendly pest management approaches for FAW.

Although several integrated pest management (IPM) options are being developed, most resource-constrained smallholder maize farmers cannot access them [17,18,19]. IPM is often labelled as knowledge-intensive, and most smallholder farmers may not have the ecological literacy to understand IPM processes, therefore limiting their adoption [18]. Moreover, most IPM tactics are unavailable owing to a lack of private sector interest, a poor regulatory and policy environment, and a lack of public incentives [17,19].

Before the invasion of FAW, farmers relied on locally available and low-cost options, including indigenous management practices. These included mechanical control (crushing egg masses and handpicking small larvae) and cultural control (intercropping maize with common edible legumes such as beans and the application of tobacco extracts, wood ash, and soils) [20] to control similar pests such as stemborers *Busseola fusca* Fuller (Lepidoptera: Noctuidae) and *Chilo partellus* Swinhoe (Lepidoptera: Pyralidae) [21]. These indigenous pest management practices have been extended to FAW and constitute essential components of IPM. Indigenous pest management practices are environmentally friendly, readily available, and affordable to most smallholder farmers [21], making them easy to implement and disseminate. Often labelled as agroecological approaches, these indigenous approaches improve crop health and promote biodiversity, providing space and alternative resources for natural enemies [21].

Previous studies have shown that sugar solutions applied to leaves attract natural enemies, such as solitary wasps and ants, and increase their foraging capabilities [21,22]. Pouring sand and wood ash into the leaf whorl or spraying with rabbit urine has also been reported to be effective against FAW larvae [21,22,23,24,25]. These strategies are believed to deter larval feeding or to desiccate the larvae of the pest.

In Malawi, the application of fish soup plus sugar is one of the indigenous practices used by smallholder farmers to protect crops against insect pests. Despite its application, its mechanism for controlling the pest remains unknown. There is a need to generate empirical data to promote the use of fish soup in other FAW-prone regions and optimise the effectiveness of the technique. Therefore, we have evaluated the efficacy of fish soup and attempted to elucidate its mode of action for controlling FAW. The study evaluated the potential of fish soup to reduce damage and attract the biodiversity of visiting insects (potentially natural enemies of FAW). The study also provides information on the active proximate compositions and volatile organic compounds present in fish soup.

## 2. Materials and Methods

### 2.1. Study Site and Study Plant

Two experiments were conducted between August 2020 and March 2021 and between June 2022 and October 2022 at the International Centre of Insect Physiology and Ecology (ICIPE), Nairobi, Kenya (latitude 1°13′ S and longitude 36°53′ E and a mean elevation of 1587 m above sea level).

SC Duma 43 was the maize (*Zea mays* L.) variety selected for the experiments, and its seeds were sourced from Kenya Seed Company (Nairobi, Kenya). This is the variety mostly grown by smallholder farmers in many regions of Kenya. Seeds were sown in a blend of growing substrates at a ratio of 2:1:1 of topsoil, compost, and sand soil placed in planting stations in the open field. One week following germination, each plant was top-dressed with 2–3 g of NPK fertiliser (Yara East Africa Limited, Nairobi, Kenya) comprising nitrogen, phosphorus, and potassium in a ratio of 17:17:17. The plants were maintained in an open field under sufficient natural light (12 L:12 D photoperiod) at a mean daily temperature range of 23–27 °C. All good agronomic practices, including watering, topdressing, and weeding, were applied. Artificial infestation of these crops was conducted following a procedure adapted from Harrison [26] and was applied when the maize plants were 3–4 weeks old. The artificial infestation was subsequently followed by application treatments. However, the natural infestation of FAW was also expected as the experiment was conducted in an open area.

### 2.2. Fall Armyworm Colony

Fall armyworm eggs and larvae were obtained from a continuous colony reared at ICIPE. The rearing of a fall armyworm colony at *icipe* is described by Tefera et al. [27]. Fall armyworm egg masses were deposited on wax/butter paper. The rearing was conducted under laboratory conditions of 25 ± 2 °C, 72 ± 3% RH, and L12:D12 photoperiod, with the larvae feeding on maize leaves.

### 2.3. Preparation of Treatments

Freshly harvested and sun-dried small pelagic fish, *Rastrineobola argentea* (Pellegrin) (locally known as “omena” in Luo, “dagaa” in Swahili, “mukene” in Luganda, and “Ndakala” in Lingala), was obtained from the Gikomba market in Nairobi, Kenya. Two kilograms of grounded fish were boiled in 5 L of water for 45 min. The soup was then cleared from the boiled fish by sieving the mixture through a 4.5 mm mesh, and the resulting soup was left to cool to room temperature in a 5 L plastic bucket. Approximately 450 g of white sugar (Kabras Sugar Mills Ltd., Kakamega, Kenya) was stirred into the fish soup to homogenise the solution. The solution was decanted, and the liquid was poured into a 5 L bucket for the experiment.

For the first experiment, a series of dilutions of 50% and 10% were prepared from the initial concentration (100%) with the addition of distilled water. Three treatments (100%, 50%, and 10% fish soup and sugar) were prepared for immediate subsequent spray, while distilled water was used as a control.

Four solutions (25% fish soup, sugar, and 25% fish soup and sugar) were prepared for use in the second experiment. The fish soup was prepared as described above, and the resulting solid residue of fish was placed separately in clean plastic cups. As a positive control, the chemical insecticide Habel™ 5 (Jiangsu United Agrochemical Co. Ltd., Nanjing, China) with Emamectin benzoate (50 g/kg) as an active ingredient was mixed with water at the recommended concentration of 0.25 g/L. The negative control consisted of distilled water for this experiment.

### 2.4. Experimental Design and Treatment Application

**Figure 1:**
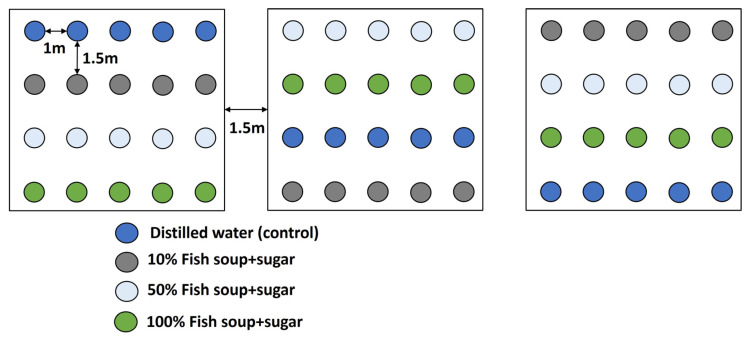
Experimental layout with maize plants and treatments.

The experimental layout followed a randomised block design. In the first experiment, four treatments (10% fish soup and sugar, 50% fish soup and sugar, 100% fish soup and sugar, and distilled water) were sprayed on the maize plants (Figure 1). Five plants were planted in four rows in a plot, at distances of 1 m between plants and 1.5 m between rows. The same design was replicated three times in three plots situated 1.5 m apart. Before applying treatments, all plants were artificially infested with egg masses of fall armyworm. The sections of wax/butter paper containing the eggs were removed and clipped onto the underside of a maize plant leaf, near the whorl, at the 3–4 leaf maize stage. Each maize plant received 30–50 eggs, and after 4 days, when most of the eggs had hatched, the plants were sprayed with four treatments, as follows: All treatments were separately loaded in a 1.5 L hand sprayer and applied to maize plants in a row designated for each treatment by spraying until dripping. Apart from the artificial infestation, maize plants were exposed to the natural infestation that usually occurs in any other maize field. Data collection began 24 h after the application of the treatments and was repeated weekly for seven weeks.

Using the same design as in the first experiment, six months later, a second experiment with five treatments was conducted. The treatments in the second experiment comprised a negative control (distilled water), 25% fish soup and sugar solution, a blend of 25% fish soup and sugar, and a positive control with the insecticide 5-Emamectin benzoate (Habel™). The optimal concentration (25%) of fish soup was based on the optimal attractiveness to the natural enemies of FAW established in the first experiment. The experiment was laid out with five rows and five plants per row in a plot. The plots were replicated three times. Each row per plot represented one treatment, which was randomly allocated. To complement natural infestation, artificial infestation of FAW larvae was carried out as described for the first experiment, when maize plants reached the 3–4 leaf stage, and repeated 5 weeks later (presented here as weeks 0 and 5, respectively). Treatment application, including negative and positive controls, was applied as described in the first experiment and repeated weekly for seven weeks.

### 2.5. Assessment of Foliar Damage and Plant Recovery

The foliar damage caused by FAW larvae feeding on crop leaves exposed to various treatments was assessed. In the first experiment, leaf (foliar) damage was evaluated using an arbitrary scale of 0% to 100%, adapted from Williams et al. [28]. Briefly, damage made by FAW larvae was visually rated based on the mark(s) on the leaves as follows: “0” mark for 0–10% damage, “1” mark for 10–20% damage, “2” mark for 20–30% damage, “3” mark for 30–40% damage, “4” mark for 40–50% damage, “5” mark for 50–60% damage, “6” mark for 60–70% damage, “7” mark for 70–80% damage, “8” mark for 80–90% damage, and “9” mark for 90–100% damage. Monitoring was carried out daily, and the foliar damage and plant recovery observations were made once weekly for two weeks. The reduction in foliar damage after two weeks was expressed as plant recovery. Recovery was calculated by the difference between the damage scores of weeks 1 and 2, using the following formulas:

*Foliar recovery* (%) = *b* − *a* (1)

where *a* is the foliar damage (%) score on week one, and *b* is the foliar damage (%) score on week two. When the calculation of recovery yielded negative values, a score of zero was recorded, which meant that damage was continuous and did not allow any plant recovery.

In the second experiment, foliar damage, but not foliar recovery, was scored for seven weeks using the protocol of Williams et al. [28].

### 2.6. Assessment of Abundance and Diversity of Visiting Insects

In the first experiment, visiting insects were collected using two trapping methods: pitfall traps and yellow sticky cards. Immediately after spraying maize plants, a pitfall trap (consisting of a 150 mL cylindrical cup, 74 mm in height with a 70 mm opening diameter) was placed adjacent to the maize stem, with the cup opening at soil level to capture potential crawling insects visiting the treated maize plants. The pitfall traps were filled with water containing a few drops of unscented multipurpose liquid detergent (Teepol^TM^, London Soap Company, London, UK) to trap crawling animals by drowning. Traps were collected after 24 h for the morphological identification of the insects. The pitfall traps were placed after applying treatments, one week after treatment, and two weeks after treatment.

To capture potential flying insects visiting the plants, 10 cm × 25 cm yellow sticky cards (Horiver^®^, Koppert Biological Systems, Nairobi, Kenya) were suspended 30 cm above the crop canopy. Four traps (two yellow and two blue) were placed along each row of maize plants in alternating order. These polystyrene sticky cards were covered with non-toxic glue on both sides. They were set after the application of treatments once a week for two weeks. The sticky cards were removed after 24 h, and the tanglefoot glue was dissolved by using kerosene to suspend the trapped insects (adapted from Muvea et al. [29]). The trapped visiting insects were labelled and arranged per treatment and organised per taxonomic group, i.e., order and family. Insects were then collected using a fine brush and transferred into glass vials containing 70% ethanol for further processing, counting, and identification using a dichotomous key up to family level.

### 2.7. Assessment of Plant Growth Parameters

The plant growth parameters (plant height and chlorophyll content) were recorded from plants sprayed with 50% fish soup and sugar solution and distilled water in the first experiment. Measurements of plant height (in cm) and chlorophyll content (SPAD value) were taken weekly for three weeks. Above-ground plant height was measured using a 1-metre tape measure. A SPAD 502 Plus Chlorophyll Meter (Honor Test Technology Co., Ltd., Shijiazhuang, China) was used to measure the amount of chlorophyll in the leaves. Weekly measurements of the chlorophyll amounts were carried out from the 3–4 leaf stage until the 24-leaf stage of the maize crop by using a chlorophyll metre (SPAD). The uppermost fully expanded leaf was selected for the first reading, and the second and third readings were of the second and third leaves under the uppermost expanded leaf in a plant. The reading for each leaf was taken at between 40 and 70% distance from the base, and readings from the three leaves were averaged.

### 2.8. Proximate Analysis and Mineral Composition of Fish Soup

The proximate analyses and mineral compositions of the three samples (25% fish soup, 25% fish soup and sugar, and fish residue) were submitted to Crop Nutrition Laboratory Services Limited (Cropnuts Ltd., Limuru, Kenya), Limuru, Kenya. The proximate compositions were estimated according to the methods of the Association of Official Analytical Chemists (AOAC) [30]. The crude protein (N × 6.25) was determined using the Kjeldahl method (method 978.04) [31] and ISO 5983-2 [32]. Crude ash, crude fat, crude fibre, and dry matter were determined using the following methods: ISO 5984 [33], ISO 6492 [34], ISO 13906 [35], and ISO 6496 [36], respectively. Mineral composition analysis was carried out using the wet chemistry Inductive Coupled Plasma—Mass Spectrometry technique (Analytik Jena GmbH + Co. KG, Jena, Germany).

Volatile organic compounds (VOCs) present in the fish soup samples and control groups were collected by capturing the headspace volatiles. Each treatment was cooled to room temperature (25 ± 2 °C) and then transferred to separate 2-L Quickfit^®^ glass chambers (Analytical Research Systems, Gainesville, FL, USA). To facilitate the collection process, activated charcoal-filtered and humidified air was circulated over the samples at a flow rate of 340 mL/min using a push–pull Gast pump (Gast Manufacturing, Benton Harbor, MI, USA). The volatiles were subsequently absorbed onto Super-Q traps (30 mg, Analytical Research Systems, Gainesville, FL, USA) at a flow rate of 170 mL/min, employing a Vacuubrand CVC2 vacuum pump (Vacuubrand, Wertheim, Germany). All VOC collections were carried out for a duration of 24 h.

Prior to collection, volatile collection traps, Super-Q^®^ traps (SQ International, Seoul, Republic of Korea), were pre-cleaned using GC-grade dichloromethane and dried with a stream of high-purity nitrogen gas provided by a nitrogen generator (Peak Scientific Instruments Ltd., model 600 cc, Renfrewshire, UK). At the end of the 24 h collection period, the Super-Q^®^ traps containing adsorbed volatiles were eluted with 200 μL of dichloromethane into 2 mL clear glass vials. Each vial was equipped with a 250 μL conical-point glass insert (Supelco, Bellefonte, PA, USA). The eluted samples were promptly subjected to analysis using gas chromatography mass spectrometry (GC-MS).

For the analyses, the extracted fish soup volatiles (1 μL) were injected, using a splitless technique, into a 7890A gas chromatograph (GC), coupled with a 5975C mass selective detector (MSD, Agilent Technologies, Santa Clara, CA, USA). The GC system was equipped with a 5%-phenyl-methylpolysiloxane (HP5 MS) low-bleed capillary column (30 m × 0.25 mm i.d., 0.25 μm; J&W, Folsom, CA, USA). The oven was programmed with the following settings: helium flow rate at 1.25 mL/min, initial oven temperature held at 35 °C for 5 min, followed by a rise at a rate of 10 °C/min to 280 °C, and then held at this temperature for 20.4 min. The MSD was operated with an ion source temperature of 230 °C and a quadrupole temperature of 180 °C. Electron impact (EI) mass spectra were obtained at 70 eV, and the fragment ions were analysed over a mass range of 40 to 550 *m*/*z* in full scan mode. A solvent delay of 3.3 min was implemented. Experiment-specific retention indices (RIs) were calculated relative to C8–C32 n-alkanes.

The relative integration of each detected peak was determined by using the ChemStation integrator and reported as the relative abundance. To eliminate contaminant peaks or peak areas originating from the adsorbent, column, or solvent, blank runs were performed on empty collection systems and analysed. Detected peaks were tentatively identified by comparing the mass spectral data with reference spectra published in MS databases, considering retention times and retention indices, and, where available, identification was made through co-injection with an authentic sample.

### 2.9. Data Analyses

The extent (percentage) of foliar damage, the percentage of plant recovery, the numbers of insect species collected on traps, plant heights, and chlorophyll contents were recorded. The insects collected on the pitfall traps and sticky cards were counted based on treatments and identified according to order, family, and genus level.

Subsequently, the means of foliar damage and percent plant recovery were subjected to analysis of variance (ANOVA).

To establish the percentage peak recovery and least damage of maize plants after treatments with 10%, 50%, and 100% fish soup and sugar solutions, the data were fitted to a modified Gompertz model [37]:

(2) $$Y\left(D\right)=Y_{Asym}exp\;\left(-exp\;\left((\frac{u_{g^{e}}}{Y_{Asym}})(\lambda_{g}-C\right)+1\;\;\right)$$

where *Y(D)* is the expected level (percentage) of recovery or damage of maize plants as a function of the concentration of fish soup and sugar solution, *Y_Asym_* is the asymptotic recovery or damage level (percentage), *λ_g_* is the inflection point of the curve (having concentration units), ug is the rate of recovery or damage, and *C* denotes the specific concentration of fish soup and sugar solution tested. To get weighted least-square estimates of these parameters, the data were fitted in the Gompertz model’s equation using the *nlsLM* function, and start values for the model to achieve convergence tolerance were based on hypothetical estimations. The corresponding least concentration (*C_op_t*) required for the peak recovery or lowest damage of the maize plant (expressed as *Y_Asym_*) was calculated from a mathematical equation where *C* was the subject of the formula.

Shannon-Weiner diversity was used to estimate the diversity of insects that visited the artificially FAW-infested maize after spraying with three fish soup and sugar solutions, alongside the control. The following parameters were assessed: The relative abundance of order was determined as follows:

*Relative abundance = n/N,* (3)

where *n* is the total number of specimens of a particular insect family, and *N* is the total number of all insect families in a particular order. Insect family richness was estimated for each treatment [38]. To assess the insect diversity, the Shannon-Weiner index (*H*′) [39] was computed using the Shannon and Weaver [40] formula:

*H’* = −⌊∑*P_i_×LN(P_i_)⌋,* (4)

where *H′* is the Diversity Index, *Pi* is the proportion of each family in the sample, and *LN (Pi)* is the natural logarithm of *Pi*. The evenness of insect families compares the similarity of the population size of each family [41].

*Evenness J’* = *H’/H_max_,* (5)

where *H_max_* is the natural log of the total number of families.

A word cloud analysis was conducted to show the abundance of different families of insects attracted by the fish soup and sugar-treated maize plants.

A probit regression was used to predict the count of different families across the different concentrations of fish soup. The predicted count was plotted against the fish soup concentration.

Datasets on plant growth parameters (plant height and chlorophyll content) were subjected to a generalised linear model (GLM). Post-hoc analyses were performed for factors showing significant differences by using Tukey’s honestly significant difference (HSD) test at *p* < 0.05. A word cloud analysis was conducted to illustrate the families of insects attracted to fish soup and sugar solution sprayed on maize plants. All statistical analyses were conducted with R Software version 4.2.3 [42].

## 3. Results

### 3.1. Effect of Fish Soup and Sugar on Foliar Damage and Recovery of FAW-Infested Maize Plants

**Figure 2:**
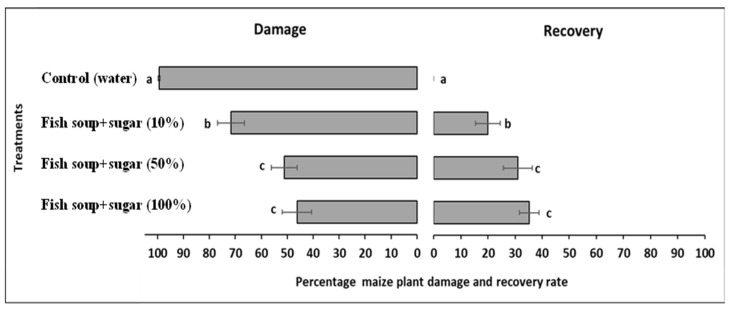
Foliar damage and recovery rate of maize plants after being sprayed with three concentrations of fish soup and sugar solution and distilled water (control). The different lowercase letters indicate significant differences of treatments according to Tukey’s honestly significant difference (HSD) test at *p* < 0.05 for foliar damage and recovery rate.

In the first experiment, maize plants that were sprayed with different doses of fish soup and sugar solutions showed significant differences in FAW foliar damage (F_3,39_ = 204.94; *p* < 0.001) and plant damage recovery (F_3,39_ = 56.60; *p* < 0.001). The percentage of foliar damage reduced significantly with increasing concentrations of fish soup and sugar solution, while the percentage of plant recovery increased with increasing concentrations of fish soup and sugar. The FAW damage results for maize plants treated with 100% fish soup and sugar, 50% fish soup and sugar, 10% fish soup and sugar, and distilled water (control) were 46.3 ± 5.6, 51.1 ± 5.0, 71.6 ± 5.2, and 99.4 ± 0.4%, respectively. Plant recovery results after application of the same treatments were 35.2 ± 3.7, 31.1 ± 5.4, 20.0 ± 4.6, and 0.0 ± 0.0%, respectively (Figure 2).

**Figure 3:**
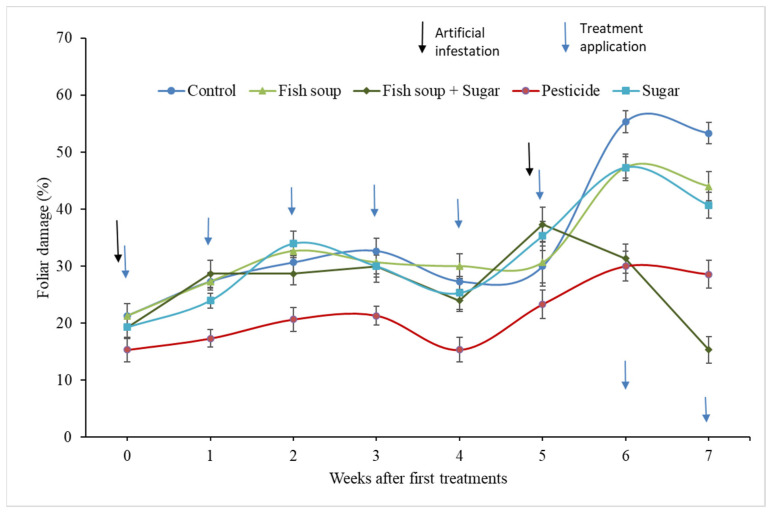
Percentage of foliar damage by fall armyworm after application of treatments. Note: Artificial infestation, treatment applications, and data collection were conducted consecutively after every 24 h in weeks 0 and 5 post-treatment applications.

In the second experiment, the foliar damage varied significantly among treatments (F_7,586_ = 61.44; *p* < 0.001) and the number of weeks after treatment application (F_4,586_ = 42.56; *p* < 0.001). Generally, the foliar damage by FAW was significantly reduced after the spray of chemical insecticides, followed by fish soup and sugar, sugar alone, and fish soup alone, with the least reduction being recorded for the control (Figure 3).

### 3.2. Estimates of Optimal Doses of Fish Soup and Sugar for the Least Foliar Damage and Peak Recovery

**Figure 4:**
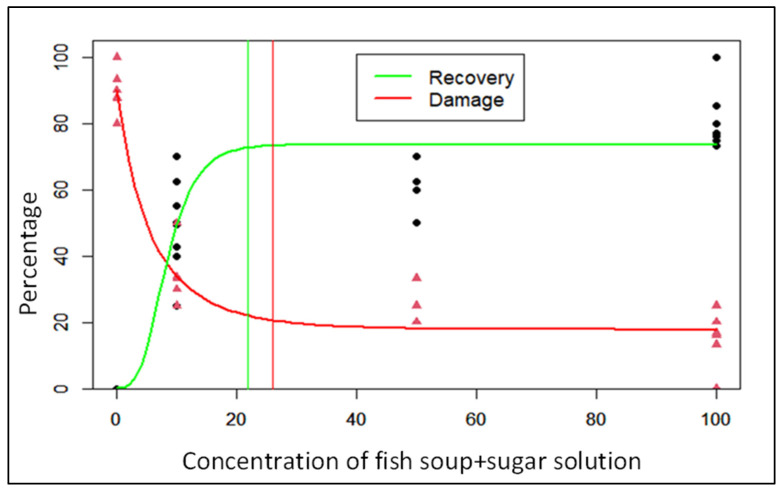
Illustration of Gompertz model’s curve of fall armyworm foliar damage and plant recovery as a function of fish concentration.

Gompertz’s model indicates that the relationships between either the percentage of FAW foliar damage or the percentage of plant recovery and concentrations of fish soup and sugar sprayed are non-linear and loosely inversely proportional (Figure 4).

The minimal FAW foliar damage and optimal plant recovery rates of maize plants treated with fish soup and sugar solutions were 17.8 ± 1.9% and 73.6 ± 2.4%, respectively (Table 1). Substitution of the Gompertz model equation indicated that concentrations of fish soup required for minimal FAW foliar damage and optimal plant recovery were 25.9 and 21.8%, respectively (Table 1).

### 3.3. Effect of Fish Soup and Sugar Solution on Maize Plant Growth Parameters

**Figure 5:**
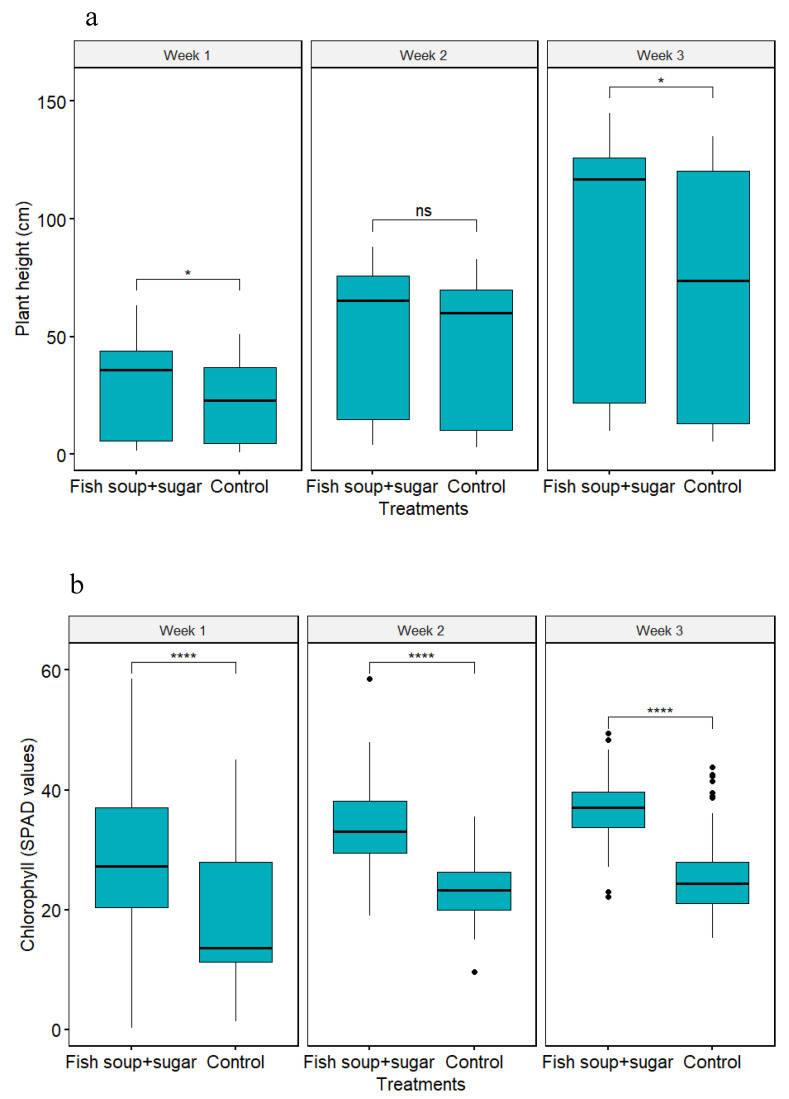
Maize plant height (**a**) and chlorophyll content (**b**) after being sprayed with a 50% concentration of fish soup and sugar and control (distilled water). ns implies no significance, while * and **** imply significance at *p* = 0.05 and *p* = 0.0001, respectively, between treatments according to the Tukey test.

Here, we compared the 50% fish soup and sugar solution with the control. The plant height differed significantly between treatments (χ^2^ = 9.60; df = 1; *p* = 0.002) and across the weeks (χ^2^ = 250.57; df = 2; *p* < 0.001). Likewise, the chlorophyll content of maize plants differed significantly between treatments (χ^2^ = 385.01; df = 1; *p* < 0.001) and across the weeks (χ^2^ = 126.86; df = 2; *p* < 0.001). Compared with the control, maize plants sprayed with a fish soup and sugar solution grew significantly faster (Figure 5a). Likewise, the chlorophyll content of maize plants sprayed with the fish soup and sugar solution was significantly higher when compared with the control (Figure 5b).

### 3.4. Diversity of Insects Visiting Fall Armyworm-Infested Maize Plants

The abundance, richness, diversity, and evenness of the different orders observed visiting FAW-infested maize after applying fish soup and sugar solutions are represented in Table 2. Hymenoptera (ants and wasps), Diptera (flies), Coleoptera (ladybirds), and Hemiptera (true bugs) were the main four taxonomic orders of insects observed. Their abundance occurred as follows: 1096, 475, 426, and 253, with diversity indices of 4.8, 5.3, 4.0, and 5.5, respectively. Maize plants treated with fish soup and sugar attracted more abundant and diverse orders of insects than the control did. Unlike the Dipterans, the abundance and diversity of Coleopterans and Hemipterans increased with the fish soup concentration. The abundance and diversity of Hymenopterans were least affected by concentrations of fish soup and sugar solutions (Table 2).

Among the Dipterans, twelve families comprising Bibionidae, Calliphoridae, Chloropidae, Diopsidae, Muscidae, Neriidae, Phoridae, Psilidae, Rhiniidae, Sepsidae, Stratiomyidae, and Syrphidae were attracted. The most abundant families in this order were Muscidae and Calliphoridae. Among the Hymenopterans, 17 families were attracted, and they comprised Agaonidae, Apidae, Bethylidae, Braconidae, Ceraphronidae, Chalcididae, Diapriidae, Dryinidae, Encyrtidae, Eulophidae, Eupelmidae, Formicidae, Halictidae, Ichneumonidae, Platygastridae, Scelionidae, and Figitidae. The most abundant families in this order were Formicidae, followed by Dryinidae. Among the Coleopterans, nine families were collected: Apionidae, Bostrichidae, Bruchidae, Chrysomelidae, Coccinellidae, Curculionidae, Mordellidae, Scarabaeidae, and Staphylinidae. Families of Chrysomelidae and Coccinellidae recorded the highest abundance.

The Hemiptera order was comparatively less diverse, with only 6 families being attracted, and the family Aphididae was the most abundant in comparison with Cercopidae, Cicadellidae, Delphacidae, Membracidae, and Miridae.

**Figure 6:**
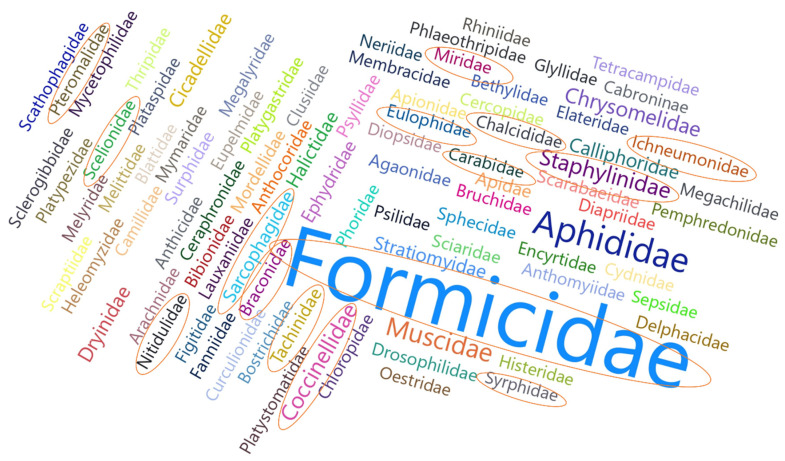
Word cloud analysis illustrating the families of insects attracted to fish soup and sugar solutions sprayed on maize plants. Families highlighted in oval shapes comprise potential natural enemies of Lepidopteran pests.

Generally, across different orders of insects observed, the most abundant insect families were Formicidae, Aphididae, Muscidae, Coccinellidae, Chrysomelidae, and Dryinidae (Figure 6).

### 3.5. Variability of Visiting Insect Community as a Function of Fish Soup Concentrations Sprayed on FAW-Infested Maize Plants

**Figure 7:**
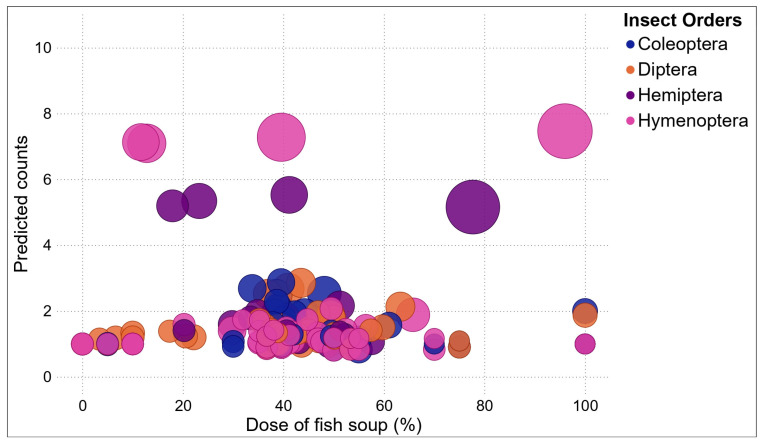
Representation of orders of insects attracted to different fish soup and sugar concentrations sprayed on the maize plants.

**Figure 8:**
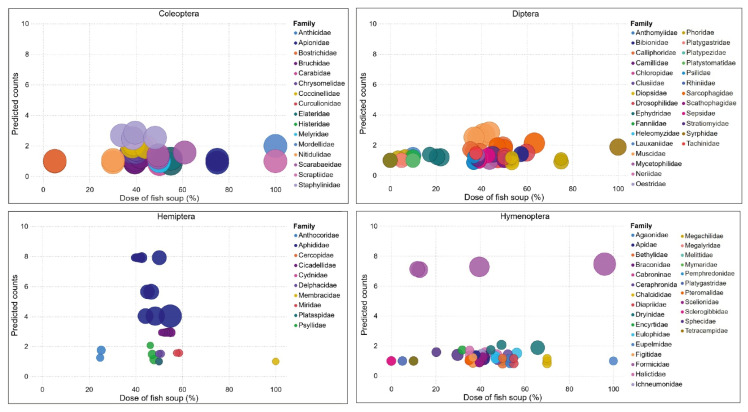
Families of insects in the order Coleoptera, Diptera, Hemiptera, and Hymenoptera attracted to different concentrations of fish soup and sugar sprayed on the maize plants. The size of the balloon plots represents the population density of insects (individuals/concentration).

A probit regression confirmed that the numbers of insects in different orders visiting treated maize plants varied across fish soup concentrations (Figure 7; Supplementary Materials). The highest frequencies of Coleopterans, Dipterans, and Hymenopterans are likely to be recorded at 30–60% concentrations of fish soup and sugar, while the frequency of Hemipterans was highest at 45–60% concentrations of fish soup and sugar (Figure 8).

### 3.6. Proximate Analysis of Fish Soup, Fish Soup and sugar, and Fish Solid Residue

The proximate analyses of fish soup, fish soup and sugar, and fish residue are presented in Table 3. We found that fish soup, fish soup and sugar, and fish solid residue contain variable proximate compounds. Except for sulphur and cobalt, the fish residue had the highest contents of proximate compounds when compared with fish soup and the blend of fish soup and sugar. Compared with fish soup alone, the blend of fish soup and sugar had higher energy levels. In general, the solid residue of fish has the highest levels of all detected proximate elements, except for sulphur and cobalt. The addition of sugar to fish soup seems to increase the levels of energy, fibre, dry matter, iron, and manganese while reducing the levels of protein, total ash, fat, calcium, potassium, magnesium, phosphorus, sulphur, boron, copper, zinc, sodium, and cobalt.

### 3.7. Volatile Organic Compounds in Fish Soup

**Figure 9:**
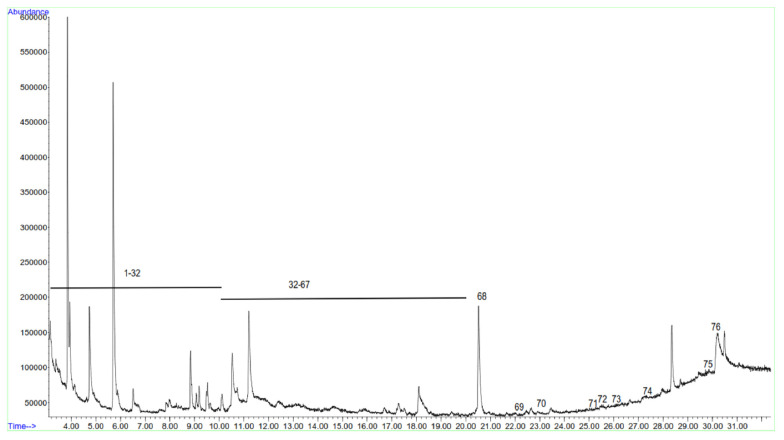
Representative total ion chromatogram of fish soup volatile organic compounds.

Figure 9 illustrates the chromatograms of different compounds detected in fish soup. GC/MS analysis of the fish soup revealed the presence of a total of 76 volatile organic compounds (VOCs) (Table 4). These compounds were categorised into different chemical classes, including alcohol (**12**), monoterpene (**11**), sesquiterpene (**10**), ketone (**7**), aldehyde (**5**), alkene (**5**), esther (**5**), amine (**3**), cyclopropane derivative (**3**), heterocyclic (**3**), phenol (**2**), spiro compound (**2**), diene (**1**), hydoxy derivative (**1**), imidazole derivative (**1**), indole (**1**), sulfoxide (**1**), thiol (**1**), and triterpene (**1**). Of particular interest, we identified 16 compounds in the fish soup that have previously been reported as insect attractants. These compounds are 5-methyl-2-heptanone, acetoin, isopentyl formate, 2E-pentenal, 1,8-nonadien-3-ol, 3Z-hexenol, 1-octen-3-ol, α-pinene, β-phellandrene, P-cymene, perpinolene, α-copaene, α-humulene, β-longipinene, 1,3-adamantanediacetamide, and 9E-octadecenoic acid.

## 4. Discussion

Our study partly confirms the assertions and claims made by Malawian smallholder farmers about the efficacy of fish soup and its potential use in integrated pest management strategies [21]. We demonstrated that FAW-infested maize plants treated with fish soup and sugar solutions at varying concentrations experienced lower foliar damage and higher recovery than the control plants did, which showed continued leaf damage with no recovery. We found that foliar damage was inversely correlated with the concentration of fish soup and sugar, while plant recovery was positively correlated with the concentration of fish soup and sugar. The effect of chemical insecticides in reducing foliar damage did not deviate much from fish soup and sugar solutions, as compared with the control.

Fish soup has multiple modes of action for managing FAW. We found that fish soup contains main plant-required macroelements (calcium, magnesium, nitrogen, phosphorus, and sodium) and microelements, such as sulphur, boron, manganese, iron, zinc, copper, and molybdenum, which can boost crop growth and health. These mineral nutrients may have promoted rapid growth (plant height) and higher chlorophyll content in fish soup-treated FAW-infested maize plants. Consequently, the improved plant growth vigour could have led to reduced pest damage and enhanced plant recovery.

Interestingly, fish soup and sugar contain energy, fats, and proteins at levels that might attract other insects, such as adult natural enemies of FAW in the maize ecosphere (Supplementary Materials). The diversity of insects attracted to maize plants treated with fish soup and sugar solutions included families of potential natural enemies (predators and parasitoids) of FAW. The diversity of insects also increased with the dose, especially between 30 and 60%, implying that the highest concentration of fish soup and sugar is not necessarily the most effective. The fish soup and sugar solutions may have emitted a complex of volatiles that triggered a high convergence of visiting insects. The solid fraction of the soup provides proteins and fats that are essential for predatory and detritivorous insects, and the sugars in the soup might also enhance the fitness of parasitoids [43,44].

Using semiochemicals that can recruit the natural enemies of agricultural pests is considered a feasible and sustainable technique for promoting biological control [45,46]. The artificial induction of natural enemies into crops infested with pests may contribute to the reduction of pest populations and plant damage. In this study, the analysis of VOCs in fish soup has revealed a wide range of compounds that potentially display different biological functions, including acting as aggregation pheromones, sex pheromones, kairomones, allomones, or mimicking gland secretions of insects [47]. Notably, specific compounds have been linked to the attraction of distinct insect species. For instance, terpenes such as β-phellandrene and (E)- and (Z)-β-ocimene attract *Closterocerus ruforum* (Krausse) (Hymenoptera: Eulophidae), an egg parasitoid of the pine sawfly *Diprion pini* [48]. (E)-β-caryophyllene is very attractive to the female wasp *Aphidius ervi* (Haliday) (Hymenoptera: Braconidae), an aphid parasitoid [49]. Kairomone lures, based on blends of monoterpenes such as α-pinene, α-phellandrene, 3-carene, and β-ocimene, have been demonstrated to recruit predators for the biological control of pests such as tomato leafminer *Tuta absoluta* and greenhouse whitefly *Trialeurodes vaporariorum* [50]. Milonas et al. [51] showed that females of *Trichogramma* spp., parasitoids of *T. absoluta*, are attracted to (Z)-3-hexen-1-ol. Furthermore, (Z)-3-hexen-1-ol, when placed in traps, attracts predators such as the bug *Orius similis* and the syrphid fly *Paragus quadrifasciatus* [52]. N-heptanal and α-pinene have been previously shown to attract *Cotesia vestalis* larval parasitoid in order to control the diamondback moth (DBM) *Plutella xylostella* larvae [53]. A blend of α-humulene, β-pinene, and (E)-3-hexen-1-ol has been demonstrated to be highly attractive to predatory lady beetles *Harmonia axyridis* (Coleoptera: Coccinellidae) and *Coccinella septempunctata* (Coleoptera: Coccinellidae) in pumpkin and wheat fields [54]. Although the current study did not assess the bioactivity of individual compounds detected, it provides evidence that fish soup VOCs contain known insect attractants, explaining the diverse insect family recruited. Further research is required to fully characterise and exploit these compounds in the management of FAW.

According to CABI [55], the Spodoptera genus is associated with 247 species of parasitoids and predators. In our study, we found that Coccinellidae was the most abundant group of the order Coleoptera, with 3 species (*Coccinella undecimpunctata* L., *Scymnus* spp., and *Cheilomenes sulphurea* (Olivier)) that prey on pests of the genus Spodoptera [55]. The Muscidae and Tachinidae families are known to include natural enemies of the genus *Spodoptera*. Some of the known parasitoids of *Spodoptera* spp. in East Africa include *Palexorista zonata* (Curran), with 12.5% parasitism [56]. Tachinidae have the highest number of natural enemies of the *Spodoptera* group in this order, with 46 parasitoid species [55].

Natural enemies in the order Hymenoptera known to be present in East Africa include *Charops ater* Szépligeti and *Campoletis* spp. from the family Ichneumonidae; *Cotesia icipe* Fernández-Triana and Fiaboe; *Chelonus curvimaculatus* Cameron; *Coccygidium luteum* (Brullé) from the family Braconidae; *Telenomus remus* (Nixon) from the family Scelionidae; and *Trichogramma chilonis* Ishii from Trichogrammatoidea [56]. In a previous study by Sisay et al. [57], *T. remus* was found to be the most dominant egg parasitoid in Kenya, causing up to 69.3% of egg parasitism, followed by *T. chilonis* (Ishii), which accounted for 20.9% of egg parasitism. *Cotesia vestalis* (Kurdjumov) was recorded as the dominant larval parasitoid in Ethiopia, Kenya, and Tanzania, with percentage parasitism ranging from 16 to 42%. In this study, large numbers of ants (Formicidae) were collected in pitfall traps and observed to prey on FAW larvae. Natural enemies of lepidopteran pests from the order Hymenoptera amount to 81 species, with families Braconidae, Ichneumonidae, Eulophidae, and Formicidae having 45, 21, 13, and 2 species, respectively [55]. Some members of hemipterans in the family Miridae, like *Deraeocoris nebulosus* (Uhler), have been reported to be predators of FAW [58]. Five species of this family are predators of Lepidopteran pests [55]. On the other hand, the Aphididae seemed less attracted to the fish soup. However, the diversity and abundance of the natural enemies of FAW may vary according to geographical areas, agronomic practices, crop type, and stage [22,56,59,60,61,62,63]. Thus, despite the diversity of the natural enemies collected, some species might be underrepresented in this study due to a lack of efficient collection methods or the geographical location of the study.

## 5. Conclusions

This study confirms the claims made by Malawian smallholder farmers about the efficacy of fish soup and its potential inclusion in IPM. The uniqueness of fish soup is that, on the one hand, it contains plant mineral nutrients and acts as foliar fertiliser to improve plant vigour and recovery. On the other hand, fish soup contains high levels of energy, fat, protein, and volatiles that attract a rich diversity of insects, including natural enemies of FAW.

Indigenous pest management practices, such as applying fish soup, should be scaled up to other African maize farmers. Such technologies are likely to be more accepted because the materials are culturally embedded in the communities, readily available, accessible, and affordable to smallholder farmers. We, therefore, recommend that further in-depth studies be undertaken in chemical ecology, agroecology, and socio-economic analysis for optimisation and product development.

## Figures and Tables

**Table 1:** Estimates of the Gompertz model parameters.

Parameters	*Y_Asym_* (±SE%)	*ug*	*λg*	*C_opt_* (%)
FAW foliar damage	17.8 ± 1.8	0.61	−5.58	25.9
Plant recovery	73.6 ± 2.4	8.07	3.54	21.8

In the Gompertz model, *Y_Asym_* is the asymptotic FAW foliar damage or plant recovery level (%), λg is the curve’s inflexion point, and ug is the damage rate or recovery rate, while *C_opt_* is the concentration of fish soup and sugar solution corresponding to *Y_Asym_* (peak recovery or least damage). SE = Standard error.

**Table 2:** Abundance and diversity of insects visiting maize plant treated with fish soup and sugar and distilled water (control).

Insect Order	Treatment Type	Abundance	Richness	Shannon-Weiner Diversity Index	Evenness
Coleoptera	Control	93	51	3.7	0.9
	Fish soup and sugar (10%)	71	41	3.4	0.9
	Fish soup and sugar (50%)	95	56	3.8	0.9
	Fish soup and sugar (100%)	149	79	4.1	0.9
Diptera	Control	60	39	3.5	0.9
	Fish soup and sugar (10%)	164	102	4.4	1.0
	Fish soup and sugar (50%)	105	68	4.0	0.9
	Fish soup and sugar (100%)	89	48	3.6	0.9
Hemiptera	Control	84	17	2.2	0.8
	Fish soup and sugar (10%)	30	14	2.5	0.9
	Fish soup and sugar (50%)	50	23	2.7	0.9
	Fish soup and sugar (100%)	79	27	3.0	0.9
Hymenoptera	Control	118	55	3.7	0.9
	Fish soup and sugar (10%)	278	93	3.9	0.9
	Fish soup and sugar (50%)	210	49	2.9	0.8
	Fish soup and sugar (100%)	400	62	3.0	0.7

**Table 3:** Proximate composition of fish soup powder.

Proximate Composition	Unit	Fish Soup	Fish Soup and sugar	Residue (Solid)	Method
Energy	MJ/kg	1.03	6.03	6.04	Calculated
Protein	%	5.35	4.13	66.6	ISO 5983-2 [32]
Total ash	%	1.00	0.79	13.00	ISO 5984 [33]
Fat	%	0.43	<0.10	12.4	ISO 6492 [34]
Fibre	%	<0.02	0.16	7.09	ISO 13906 [35]
Dry matter	%	6.59	36.8	40.7	ISO 6496 [36]
Calcium	%	0.013	0.008	3.980	ICP-MS
Potassium	%	0.24	0.16	0.54	ICP-MS
Magnesium	%	0.0067	0.0042	0.1700	ICP-MS
Phosphorous	%	0.11	0.07	2.42	ICP-MS
Sulphur	%	0.075	0.051	0.740	ICP-MS
Boron	ppm	0.029	−0.014	0.72	ICP-MS
Molybdenum	ppm	<0.10	<0.10	0.30	ICP-MS
Iron	ppm	7.95	8.57	131	ICP-MS
Copper	ppm	−0.32	−0.4	5.70	ICP-MS
Zinc	ppm	2.72	1.15	273	ICP-MS
Manganese	ppm	0.28	0.73	26.0	ICP-MS
Sodium	ppm	463	286	1450	ICP-MS
Cobalt	ppm	0.016	0.012	<0.01	ICP-MS

ISO = International Organization for Standardization, ICP-MS = Inductive Coupled Plasma—Mass Spectrometry

**Table 4:** Volatile organic compounds detected in fish soup headspace.

Peak no.	RT	RI	Compound Name	Chemical Class	Abundance *
1	3.15 b	–	5-methyl-2-heptanone	Ketone	0.4 ± 0.11
2	3.18 b	–	3-thietanol	Thiol	0.1 ± 0.01
3	3.33 b	–	Acetoin	Ketone	2.3 ± 0.94
4	3.43 a	–	Ethyl propanoate	Esther	1.0 ± 0.37
5	3.74 b	579	3,7-dimethyl nonane	Alkane	0.2 ± 0.01
6	3.85 b	628	Isopentyl formate	Esther	3.1 ±0.56
7	3.89 a	644	3-methyl-1-butanol	Alcohol	41.4 ± 0.91
8	3.94 a	664	2-methyl-1-butanol	Alcohol	3.8 ± 2.26
9	4.13 b	704	7-octen-2-one	Ketone	0.3 ± 0.05
10	4.47 b	716	(E)-2-pentenal	Aldehyde	0.1 ± 0.03
11	4.73 a	725	Pentanol	Alcohol	3.0 ± 1.62
12	4.88 b	731	Z-2-penten-1-ol	Alcohol	0.5 ± 0.17
13	5.35 b	748	3-hexanone	Ketone	0.1 ± 0.04
14	5.70 a	761	Hexanal	Aldehyde	6.9 ± 1.54
15	5.88 b	767	6-methyl-2-heptanol	Alcohol	0.5 ± 0.07
16	6.75 b	799	1,8-nonadien-3-ol	Alcohol	0.1 ± 0.04
17	7.42 a	825	(Z)-3-hexen-1-ol	Alcohol	0.6 ± 0.09
18	7.98 b	847	3-esthyl-2-methyl-2-pentene	Alkene	0.4 ± 0.11
19	8.32 a	860	2-heptanone	Ketone	0.2 ± 0.01
20	8.55 b	869	5-hepten-2-one	Ketone	0.2 ± 0.02
21	8.56 a	869	Heptanal	Aldehyde	0.2 ± 0.02
22	8.84 b	880	1,2,3,4,5-pentamethyl cyclopentane	Cycloalkane	0.9 ± 0.15
23	8.97 b	885	m-mentha-4,8-diene, (1s,3s)-(+)-	Monoterpene	0.3 ± 0.06
24	9.07 a	889	α-thujene	Monoterpene	0.4 ± 0.12
25	9.19 a	894	α-pinene	Monoterpene	0.7 ± 0.16
26	9.32 b	899	2-(3-hydroxy-propyl)-cyclohexanol	Alcohol	0.1 ± 0.01
27	9.48 b	906	ethyl 3-methylbutylbutanoate	Esther	0.2 ± 0.09
28	9.63 b	913	3,3-dimethyl-2-pentanol	Alcohol	0.4 ± 0.06
29	9.63 b	913	cyclohexane, (1,2,2-trimethylbutyl)-	Cycloalkane	0.3 ± 0.09
30	9.83 b	922	11-oxa-dispiro [4.0.4.1] undecan-1-ol	Spiro compound	0.2 ± 0.02
31	9.86 b	923	β-hydroxyImidazole-5-propionic acid	Imidazole derivative	0.3 ± 0.04
32	10.08 a	933	δ-2-carene	Monoterpene	1.0 ± 0.50
33	10.21 b	939	3,5,5-trimethyl-2-hexene	Alkene	0.8 ± 0.19
34	10.31 a	943	1-octen-3-ol	Alcohol	0.9 ± 0.34
35	10.49 a	951	Myrcene	Monoterpene	2.2 ± 0.39
36	10.73 a	962	γ-terpinene	Monoterpene	0.7 ± 0.26
37	11.13 a	980	p-cymene	Monoterpene	0.6 ± 0.24
38	11.21 a	984	β-phellandrene	Monoterpene	3.2 ± 0.71
39	11.41 b	993	(E)-1,2-cyclopropanedicarboxylic acid	Cyclopropane derivative	0.4 ± 0.16
40	11.65 a	1005	(Z)-β-ocimene	Monoterpene	0.1 ± 0.08
41	11.77 a	1012	Benzeneacetaldehyde	Aldehyde	0.4 ± 0.10
42	12.27 a	1042	Dimethyl sulfoxide	Sulfoxide	0.2 ± 0.10
43	12.32 a	1045	Terpinolene	Monoterpene	0.6 ± 0.23
44	12.53 b	1058	1-cyclohexene-1-methanol	Alcohol	0.4 ± 0.07
45	12.65 b	1065	2,6-dimethyl cyclohexanol	Alcohol	0.5 ± 0.17
46	13.31 b	1105	3,4-dimethyl-2-cyclopenten-1-one	Ketone	0.4 ± 0.16
47	13.85 b	1136	2-butyl furan	Heterocyclic compound	0.2 ± 0.04
48	14.07 b	1149	m-aminobenzamidine	Amine	0.4 ± 0.15
49	14.10 b	1151	2,5-diethylphenol	Phenol	0.4 ± 0.17
50	14.18 b	1156	1-methylenespiro [2.4] heptan-4-one	Spiro compound	0.1 ± 0.02
51	14.50 b	1175	Methyl 2-methylpentanoate	Esther	1.0 ± 0.38
52	15.65 a	1248	Terpinen-4-ol	Monoterpene alcohol	0.3 ± 0.12
53	16.30 a	1291	α-copaene	Sesquiterpene	0.2 ± 0.09
54	16.68 b	1318	α-cubebene	Sesquiterpene	0.4 ± 0.11
55	17.26 b	1360	longifolene	Sesquiterpene	0.5 ± 0.07
56	17.39 b	1369	(Z)-muurola-3,5-diene	Diene	0.3 ± 0.08
57	17.48 b	1376	α-guaiene	Sesquiterpene	0.5 ± 0.09
58	17.71 a	1392	α-humulene	Sesquiterpene	0.2 ± 0.03
59	17.97 a	1412	ɣ-muurolene	Sesquiterpene	0.1 ± 0.05
60	18.08 b	1420	Aromadendrene	Sesquiterpene	1.2 ± 0.82
61	18.09 a	1421	β-longipinene	Sesquiterpene	1.1 ± 0.51
62	18.26 b	1434	α-muurolene	Sesquiterpene	0.2 ± 0.11
63	18.36 a	1442	2,5-bis(1,1-dimethylethyl) phenol	Phenol	0.3 ± 0.12
64	19.12 b	1400	5-nitrothiophene-2-aldehyde	Aldehyde	0.1 ± 0.03
65	19.39 b	1422	Sulphurous acid, pentyl undecyl ester	Esther	0.2 ± 0.05
66	20.18 b	1488	(E)-longipinane	Sesquiterpene	2.5 ± 1.85
67	20.43 b	1603	1-pentadecene	Alkane	0.2 ± 0.05
68	20.51 a	1600	hexadecane	Alkane	2.9 ± 0.23
69	22.43 b	1669	7-methoxy-1H-indole	Indole	0.1 ± 0.02
70	23.42 b	1905	2-methyl benzothiazole	Heterocyclic compound	0.0 ± 0.01
71	25.26 b	2103	1,3-adamantanediacetamide	Amide	0.1 ± 0.04
72	25.76 b	2158	9(E)-octadecenoic acid	Fatty acid	0.1 ± 0.02
73	26.32 b	2220	2-hydroxydesmethylimipramine	Hydoxy derivative	0.1 ± 0.02
74	27.24 b	2331	2-ethylacridine	Heterocyclic compound	0.1 ± 0.08
75	30.25 b	2707	Octadecanamide	Amide	0.6 ± 0.38
76	30.49 a	2734	Squalene	Triterpene	2.7 ± 1.31

Peak no. = number of peaks representing each compound. RT = retention time in minutes. RI = retention index for HP-5 column using a homologous series of n-alkanes and a linear GC ramp program. a compound identification via authentic standard. b compounds tentatively identified by RI match to only one column and published library. * relative abundance in percentage (%) from GC-MS peak integration with standard error.

## Data Availability

Datasets related to this research are available from the corresponding author upon reasonable request.

## Supplementary Materials

The following supporting information can be downloaded at: https://www.mdpi.com/article/10.3390/life14020180/s1, Figure S1: Frequency of families of visiting insects from four orders attracted to maize plants sprayed with fish soup and sugar treatments.

## References

[1] Prasanna B., Huesing J., Eddy R., Peschke V. (2018). Fall Armyworm in Africa: A Guide for Integrated Pest Management.

[2] Goergen G., Kumar P.L., Sankung S.B., Togola A., Tamò M. (2016). First report of outbreaks of the fall armyworm *Spodoptera frugiperda* (J E Smith) (Lepidoptera, Noctuidae), a new alien invasive pest in West and Central Africa. PLoS ONE.

[3] Montezano D.G., Sosa-Gómez D.R., Roque-Specht V.F. (2018). Host plants of *Spodoptera frugiperda* (Lepidoptera: Noctuidae) in the Americas. Afr. Entomol..

[4] Colborn T. (1995). Pesticides–How research has succeeded and failed to translate science into policy: Endocrinological effects on wildlife. Environ. Health Perspect..

[5] Crowe A.S., Booty W.G. (1995). A multi-level assessment methodology for determining the potential for groundwater contamination by pesticides. Environ. Monit. Assess..

[6] Assefa F., Ayalew D. (2019). Status and control measures of fall armyworm (*Spodoptera frugiperda*) infestations in maize fields in Ethiopia: A review. Cogent Food Agric..

[7] Timilsena P.B., Niassy S., Kimathi E., Abdel-Rahman E.M., Seidl-Adams I., Wamalwa M., Tonnang H.E., Ekesi S., Hughes D.P., Rajotte E.G. (2022). Potential distribution of fall armyworm in Africa and beyond, considering climate change and irrigation patterns. Sci. Rep..

[8] Senay S.D., Pardey P.G., Chai Y., Doughty L., Day R. (2022). Fall armyworm from a maize multi-peril pest risk perspective. Front. Insect Sci..

[9] Day R., Abrahams P., Bateman M., Beale T., Clottey V., Cock M., Colmenarez Y., Corniani N., Early R., Godwin J. (2017). Fall Armyworm: Impacts and Implications for Africa. Outlooks Pest Manag..

[10] Baudron F., Zaman-Allah M.A., Chaipa I., Chari N., Chinwada P. (2019). Understanding the factors influencing fall armyworm (*Spodoptera frugiperda* J.E. Smith) damage in African smallholder maize fields and quantifying its impact on yield. A case study in Eastern Zimbabwe. Crop. Prot..

[11] Belay D.K., Huckaba R.M., Foster J.E. (2012). Susceptibility of the Fall Armyworm, *Spodoptera frugiperda* (Lepidoptera: Noctuidae), at Santa Isabel, Puerto Rico, to Different Insecticides. Fla. Èntomol..

[12] Kumela T., Simiyu J., Sisay B., Likhayo P., Mendesil E., Gohole L., Tefera T. (2018). Farmers’ knowledge, perceptions, and management practices of the new invasive pest, fall armyworm (*Spodoptera frugiperda*) in Ethiopia and Kenya. Int. J. Pest Manag..

[13] Asare-Nuamah P. (2022). Smallholder farmers’ adaptation strategies for the management of fall armyworm (*Spodoptera frugiperda*) in rural Ghana. Int. J. Pest Manag..

[14] Abrahams P., Beale T., Cock M., Corniani N., Day R., Godwin J., Murphy S., Richards G., Vos J. (2017). Fall Armyworm Status. Impacts and Control Options in Africa: Preliminary Evidence Note 14. https://www.cabi.org/Uploads/isc/Dfid%20Faw%20Inception%20Report04may2017final.pdf.

[15] Adamczyk J.J., Leonard B.R., Graves J.B. (1999). Toxicity of Selected Insecticides to Fall Armyworms (Lepidoptera: Noctuidae) in Laboratory Bioassay Studies. Fla. Èntomol..

[16] Togola A., Meseka S., Menkir A., Badu-Apraku B., Boukar O., Tamò M., Djouaka R. (2018). Measurement of Pesticide Residues from Chemical Control of the Invasive *Spodoptera frugiperda* (Lepidoptera: Noctuidae) in a Maize Experimental Field in Mokwa, Nigeria. Int. J. Environ. Res. Public Health.

[17] Niassy S., Murithii B., Omuse E.R., Kimathi E., Tonnang H., Ndlela S., Mohamed S., Ekesi S. (2022). Insight on Fruit Fly IPM Technology Uptake and Barriers to Scaling in Africa. Sustainability.

[18] Deguine J.-P., Aubertot J.-N., Flor R.J., Lescourret F., Wyckhuys K.A., Ratnadass A. (2021). Integrated pest management: Good intentions, hard realities. A review. Agron. Sustain. Dev..

[19] Srinivasan R., Tamò M., Subramanian S. (2022). The case for integrated pest management in Africa: Transition from a pesticide-based approach. Curr. Opin. Insect Sci..

[20] Hruska A.J. (2019). Fall armyworm (*Spodoptera frugiperda*) management by smallholders. CAB Rev..

[21] Harrison R.D., Thierfelder C., Baudron F., Chinwada P., Midega C., Schaffner U., van den Berg J. (2019). Agro-ecological options for fall armyworm (*Spodoptera frugiperda* JE Smith) management: Providing low-cost, smallholder friendly solutions to an invasive pest. J. Environ. Manag..

[22] Georgiev G. (2005). Bioecological characteristics of *Bracon intercessor* Nees (Hymenoptera: Braconidae) as a parasitoid of the poplar clearwing moth, *Paranthrene tabaniformis* (Rott.) (Lepidoptera: Sesiidae) in Bulgaria. J. Pest Sci..

[23] FAO (2018). Integrated Management of the Fall Armyworm on Maize a Guide for Farmer Field Schools in Africa. http://www.fao.org/faostat/en/#data/FBS.

[24] Ibrahim N.D., Audu A., Dike M.C., Lawal M. (2012). Effect of raw diatomaceous earth and plant powders on *Callosobruchus sub-innotatus* infesting *Bambara groundnut* seeds. Sci. J. Pure Appl. Sci..

[25] Kemunto D., Omuse E.R., Mfuti D.K., Tamiru A., Hailu G., Rwiza I., Belayneh Y.T., Subramanian S., Niassy S. (2022). Effect of rabbit urine on the larval behavior, larval mortality, egg hatchability, adult emergence and oviposition preference of the fall armyworm (*Spodoptera frugiperda* J.E. Smith). Agriculture.

[26] Harrison F.P. (1986). Oviposition and Subsequent Infestation of Corn by Fall Armyworm (Lepidoptera: Noctuidae). Fla. Èntomol..

[27] Tefera T., Goftishu M., Ba M., Muniappan R. (2019). A Guide to Biological Control of Fall Armyworm in Africa Using Egg Parasitoids.

[28] Williams W.P., Buckley P.M., Daves C.A. (2006). Identifying resistance in corn to southwestern corn borer (Lepidoptera: Cram-bidae), fall armyworm (Lepidoptera: Noctuidae), and corn earworm (Lepidoptera; Noctuidae). J. Agric. Urban Entomol..

[29] Muvea A.M., Waiganjo M.M., Kutima H.L. (2014). Attraction of pest thrips (Thysanoptera: Thripidae) infesting French beans to coloured sticky traps with Lurem-TR and its utility for monitoring thrips populations. Int. J. Trop. Insect Sci..

[30] AOAC (2005). Official Methods of Analysis, 18th ed.

[31] Latimer G.W. (2016). Official Methods of Analysis of AOAC International.

[32] Animal Feeding Stuffs—Determination of Nitrogen Content and Calculation of Crude Protein Content.

[33] Animal Feeding Stuffs—Determination of Crude Ash.

[34] Animal Feeding Stuffs—Determination of Fat Content.

[35] Animal Feeding Stuffs—Determination of Acid Detergent Fibre (ADF) and Acid Detergent Lignin (ADL) Contents.

[36] Animal Feeding Stuffs—Determination of Moisture and Other Volatile Matter Content.

[37] Ritz C. (2009). Toward a unified approach to dose–response modeling in ecotoxicology. Environ. Toxicol. Chem..

[38] Dettmers R., Buehler D.A., Bartlett J.G., Klaus N.A. (1999). Influence of Point Count Length and Repeated Visits on Habitat Model Performance. J. Wildl. Manag..

[39] Hutcheson K. (1970). A test for comparing diversities based on the shannon formula. J. Theor. Biol..

[40] Shannon C.E., Weaver W. (1949). The Mathematical Theory of Communication.

[41] Kiros S., Afework B., Legese K. (2018). A preliminary study on bird diversity and abundance from Wabe fragmented forests around Gubre subcity and Wolkite town, Southwestern Ethiopia. Int. Int. J. Avian Wildl. Biol..

[42] R Core Team (2022). R: A Language and Environment for Statistical Computing, R Foundation for Statistical Computing.

[43] Bortolotto O.C. (2014). Sugar solution treatment to attract natural enemies and its impact on fall armyworm (*Spodoptera frugiperda*) in maize fields. Interciencia.

[44] Canas L.A., O’Neil R.J. (1998). Applications of sugar solutions to maize, and the impact of natural enemies on Fall Armyworm. Int. J. Pest Manag..

[45] James D.G. (2005). Further Field Evaluation of Synthetic Herbivore-Induced Plan Volatiles as Attractants for Beneficial Insects. J. Chem. Ecol..

[46] Cook S.M., Khan Z.R., Pickett J.A. (2007). The Use of Push-Pull Strategies in Integrated Pest Management. Annu. Rev. Èntomol..

[47] Pheromones Database: Terpenes, Propanogenins, and Others. https://lepipheromone.sakura.ne.jp/mepdb_eng.html.

[48] McCormick A.C., Unsicker S.B., Gershenzon J. (2012). The specificity of herbivore-induced plant volatiles in attracting herbivore enemies. Trends Plant Sci..

[49] Sasso R., Iodice L., Digilio M.C., Carretta A., Ariati L., Guerrieri E. (2007). Host-locating response by the aphid parasitoid *Aphidius ervito* tomato plant volatiles. J. Plant Interact..

[50] Ayelo P.M., Yusuf A.A., Pirk C.W.W., Chailleux A., Mohamed S.A., Deletre E. (2021). Terpenes from herbivore-induced tomato plant volatiles attract *Nesidiocoris tenuis* (Hemiptera: Miridae), a predator of major tomato pests. Pest Manag. Sci..

[51] Milonas P.G., Anastasaki E., Partsinevelos G. (2019). Oviposition-Induced Volatiles Affect Electrophysiological and Behavioral Responses of Egg Parasitoids. Insects.

[52] Yu H., Zhang Y., Wu K., Gao X.W., Guo Y.Y. (2008). Field-Testing of Synthetic Herbivore-Induced Plant Volatiles as Attractants for Beneficial Insects. Environ. Èntomol..

[53] Conboy N.J., McDaniel T., George D., Ormerod A., Edwards M., Donohoe P., Gatehouse A.M.R., Tosh C.R. (2020). Volatile Organic Compounds as Insect Repellents and Plant Elicitors: An Integrated Pest Management (IPM) Strategy for Glasshouse Whitefly (*Trialeurodes vaporariorum*). J. Chem. Ecol..

[54] Zhao J., Wang Z., Li Z., Shi J., Meng L., Wang G., Cheng J., Du Y. (2020). Development of lady beetle attractants from floral volatiles and other semiochemicals for the biological control of aphids. J. Asia-Pac. Èntomol..

[55] CABI (2017). Spodoptera Frugiperda (Fall Armyworm) Invasive Species Compendium. http://www.cabi.org/isc/datasheet/29810.

[56] Sisay B., Simiyu J., Malusi P., Likhayo P., Mendesil E., Elibariki N., Wakgari M., Ayalew G., Tefera T. (2018). First report of the fall armyworm, *Spodoptera frugiperda* (Lepidoptera: Noctuidae), natural enemies from Africa. J. Appl. Èntomol..

[57] Sisay B., Simiyu J., Mendesil E., Likhayo P., Ayalew G., Mohamed S., Subramanian S., Tefera T. (2019). Fall Armyworm, *Spodoptera frugiperda* Infestations in East Africa: Assessment of Damage and Parasitism. Insects.

[58] Wheeler A.G., Stinner B.R., Henry T.J. (1975). Biology and Nymphal Stages of *Deraeocoris nebulosus* (Hemiptera: Miridae), a Predator of Arthropod Pests on Ornamentals. Ann. Èntomol. Soc. Am..

[59] Hay-Roe M.M., Meagher R.L., Nagoshi R.N., Newman Y. (2016). Distributional patterns of fall armyworm parasitoids in a corn field and a pasture field in Florida. Biol. Control..

[60] Ruíz-Nájera R.E., Molina-Ochoa J., Carpenter J.E., Espinosa-Moreno J.A., Ruíz-Nájera J.A., Lezama-Gutiérrez R., Foster J.E. (2007). Survey for Hymenopteran and Dipteran Parasitoids of the Fall Armyworm (Lepidoptera: Noctuidae) in Chiapas, Mexico. J. Agric. Urban Èntomol..

[61] Kenis M., du Plessis H., Van den Berg J., Ba M.N., Goergen G., Kwadjo K.E., Baoua I., Tefera T., Buddie A., Cafà G. (2019). *Telenomus remus*, a Candidate Parasitoid for the Biological Control of *Spodoptera frugiperda* in Africa, is already Present on the Continent. Insects.

[62] Agboyi L.K., Goergen G., Beseh P., Mensah S.A., Clottey V.A., Glikpo R., Buddie A., Cafà G., Offord L., Day R. (2020). Parasitoid Complex of Fall Armyworm, *Spodoptera frugiperda*, in Ghana and Benin. Insects.

[63] Koffi D., Kyerematen R., Eziah V.Y., Agboka K., Adom M., Goergen G., Meagher R.L. (2020). Natural Enemies of the Fall Armyworm, *Spodoptera frugiperda* (J.E. Smith) (Lepidoptera: Noctuidae) in Ghana. Fla. Èntomol..

