# Supplementary material for: Validating Indigenous Farmers’ Practice in the Management of the Fall Armyworm Spodoptera frugiperda (J. E. Smith) in Maize Cropping Systems in Africa
Source: Life (Basel). 2024 Jan 25;14(2):180. doi: 10.3390/life14020180 (PMC10890150; doi:10.3390/life14020180)
Supplement: Supplementary file 1 [file life-14-00180-s001.zip › life-2737674-supplementary.pdf]

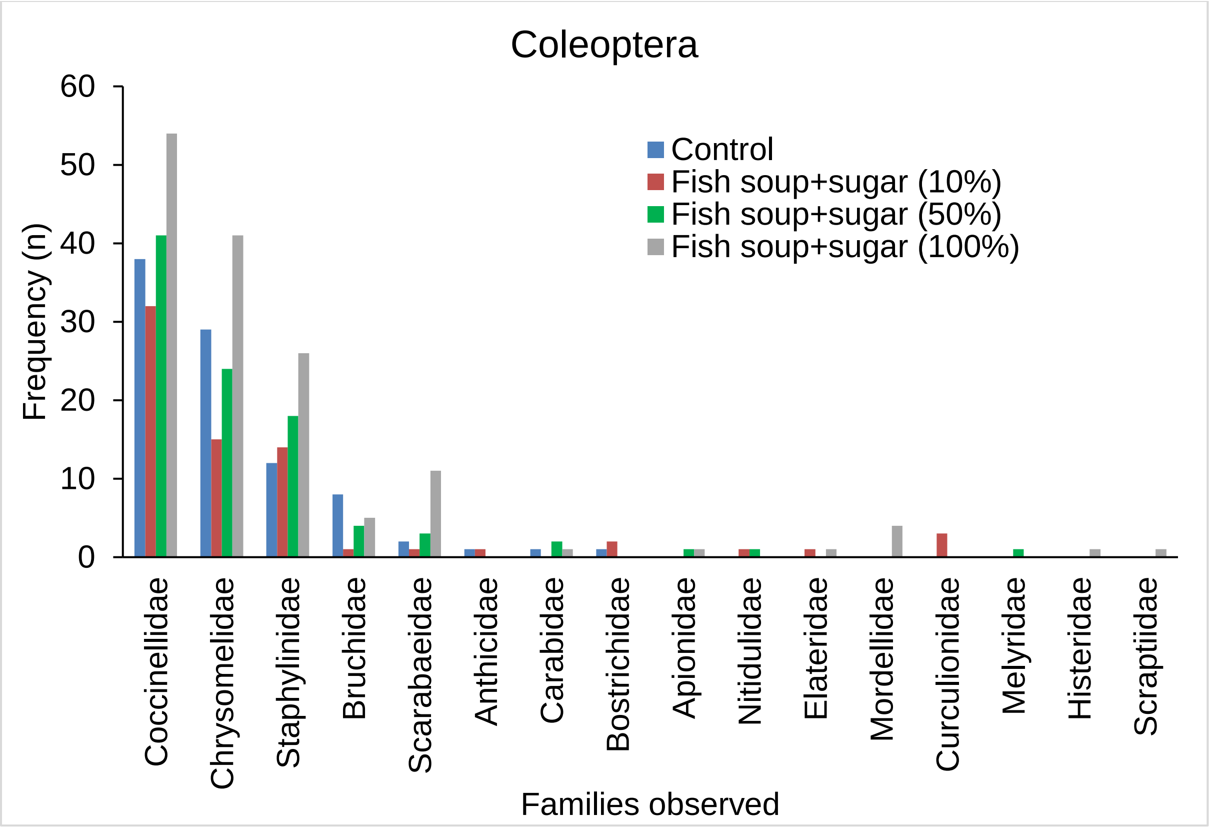
Coleoptera
Frequency (n)
0
10
20
30
40
50
60
Control
Fish soup+sugar (10%)
Fish soup+sugar (50%)
Fish soup+sugar (100%)
Coccinellidae
Chrysomelidae
Staphylinidae
Bruchidae
Scarabaeidae
Anthicidae
Carabidae
Bostrichidae
Apionidae
Nitidulidae
Elateridae
Mordellidae
Curculionidae
Melyridae
Histeridae
Scraptiidae
Families observed

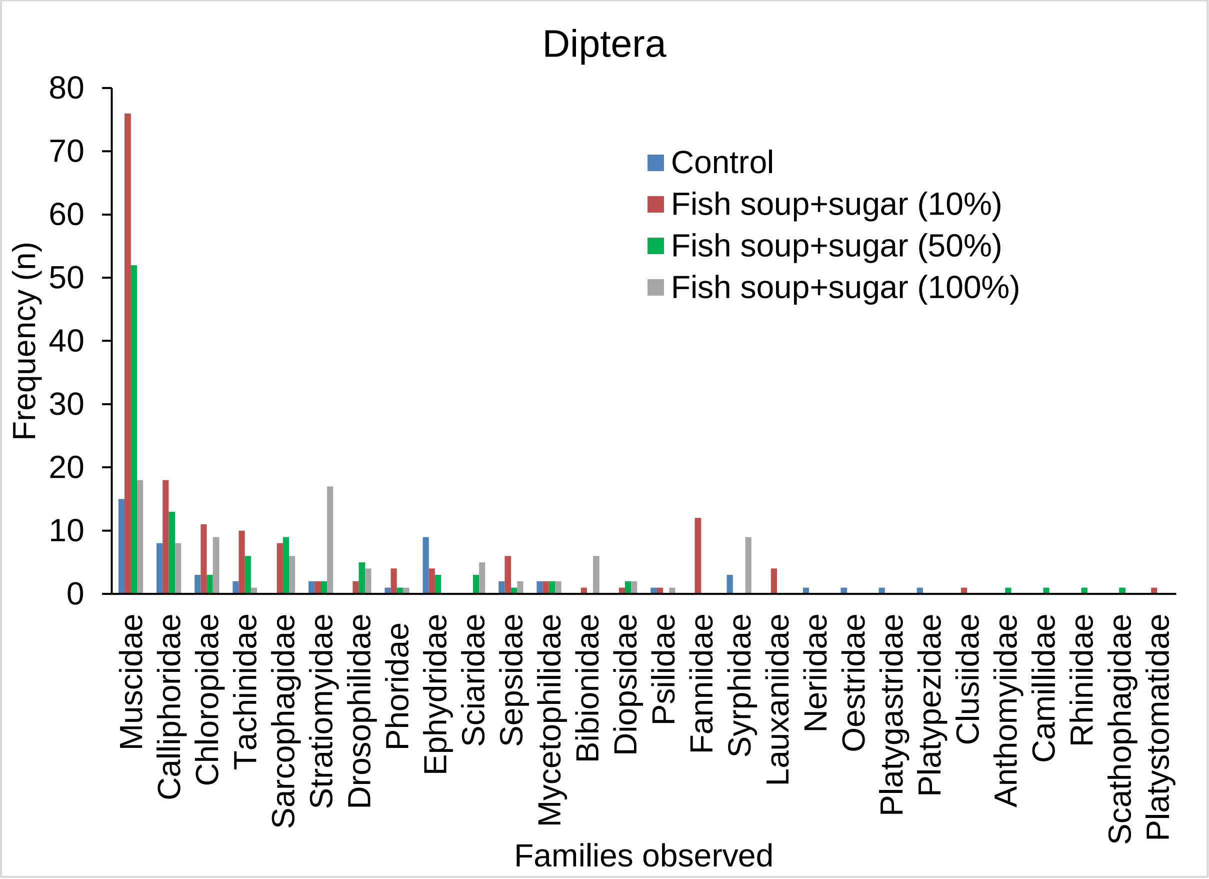
Diptera
Frequency (n)
0
10
20
30
40
50
60
70
80
Control
Fish soup+sugar (10%)
Fish soup+sugar (50%)
Fish soup+sugar (100%)
Muscidae
Calliphoridae
Chloropidae
Tachinidae
Sarcophagidae
Stratiomyidae
Drosophilidae
Phoridae
Ephydridae
Sciaridae
Sepsidae
Mycetophilidae
Bibionidae
Diopsidae
Psilidae
Fanniidae
Syrphidae
Lauxaniidae
Neriidae
Oestridae
Platygastridae
Platypezidae
Clusiidae
Anthomyiidae
Camillidae
Rhiniidae
Scathophagidae
Platystomatidae
Families observed

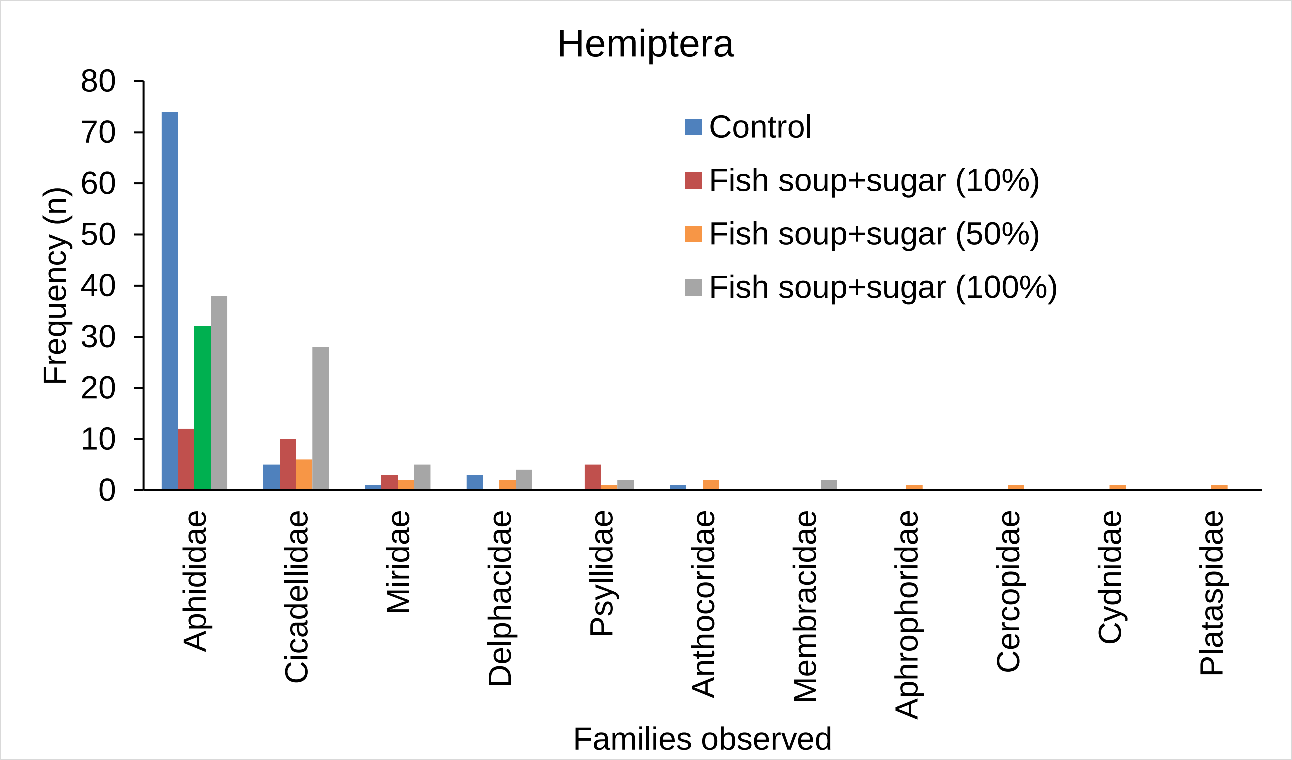

Hemiptera
Frequency (n)
80
70
60
50
40
30
20
10
0
Control
Fish soup+sugar (10%)
Fish soup+sugar (50%)
Fish soup+sugar (100%)
Aphididae
Cicadellidae
Miridae
Delphacidae
Psyllidae
Anthocoridae
Membracidae
Aphrophoridae
Cercopidae
Cydnidae
Plataspidae
Families observed

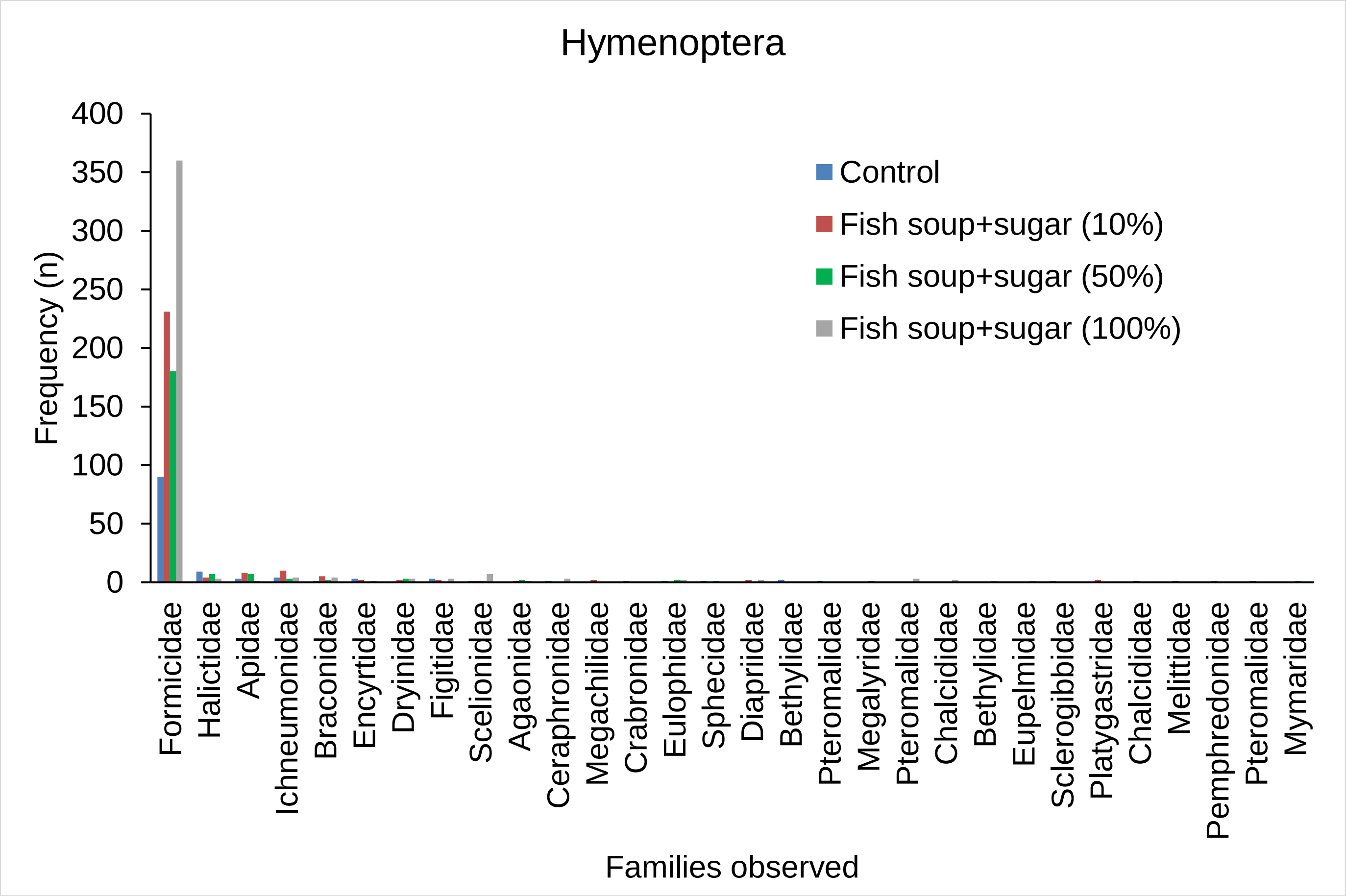

Hymenoptera
Frequency (n)
400
350
300
250
200
150
100
50
0
Control
Fish soup+sugar (10%)
Fish soup+sugar (50%)
Fish soup+sugar (100%)
Formicidae
Halictidae
Apidae
Ichneumonidae
Braconidae
Encyrtidae
Dryinidae
Figitidae
Scelionidae
Agaonidae
Ceraphronidae
Megachilidae
Crabronidae
Eulophidae
Sphecidae
Diapriidae
Bethylidae
Pteromalidae
Megalyridae
Pteromalidae
Chalcididae
Bethylidae
Eupelmidae
Sclerogibbidae
Platygastridae
Chalcididae
Melittidae
Pemphredonidae
Pteromalidae
Mymaridae
Families observed

Figure S1: Frequency of families of visiting insects from four orders attracted to maize plants sprayed with fish soup and sugar treatments.
